# Phase transition induced strain in ZnO under high pressure

**DOI:** 10.1038/srep24958

**Published:** 2016-05-13

**Authors:** Xiaozhi Yan, Haini Dong, Yanchun Li, Chuanlong Lin, Changyong Park, Duanwei He, Wenge Yang

**Affiliations:** 1 Institute of Atomic and Molecular Physics, Sichuan University, Chengdu 610065, P. R. China; 2 Center for High Pressure Science and Technology Advanced Research (HPSTAR), Shanghai 201203, P. R. China; 3 Key Laboratory of High-temperature and High-pressure Study of the Earth’s Interior, Institute of Geochemistry, Chinese Academy of Sciences, Guiyang, Guizhou 550081, China; 4 Beijing Synchrotron Radiation Laboratory, Institute of High Energy Physics, Chinese Academy of Sciences, Beijing 100039, P. R. China; 5 HPCAT, Carnegie Institution of Washington, 9700 South Cass Avenue, Argonne, Illinois 60439, US; 6 Institute of Fluid Physics and National Key Laboratory of Shockwave and Detonation Physic, China Academy of Engineering Physics, Mianyang 621900, P. R. China; 7 High Pressure Synergetic Consortium (HPSynC), Geophysical Laboratory, Carnegie Institution of Washington, Argonne, Illinois 60439, USA

## Abstract

Under high pressure, the phase transition mechanism and mechanical property of material are supposed to be largely associated with the transformation induced elastic strain. However, the experimental evidences for such strain are scanty. The elastic and plastic properties of ZnO, a leading material for applications in chemical sensor, catalyst, and optical thin coatings, were determined using *in situ* high pressure synchrotron axial and radial x-ray diffraction. The abnormal elastic behaviors of selected lattice planes of ZnO during phase transition revealed the existence of internal elastic strain, which arise from the lattice misfit between wurtzite and rocksalt phase. Furthermore, the strength decrease of ZnO during phase transition under non-hydrostatic pressure was observed and could be attributed to such internal elastic strain, unveiling the relationship between pressure induced internal strain and mechanical property of material. These findings are of fundamental importance to understanding the mechanism of phase transition and the properties of materials under pressure.

Pressure induced phase transition has been considered as an important and extensively used approach in designing new materials^1^,^2^,^3^,^4^,^5^ and in geoscientific research^6^,^7^. For instance, under high pressure and high temperature soft graphite and carbon transform into diamond, the hardest known bulk material^8^,^9^; olivine in the deep earth transforms to spinel and result in the abnormal seismic velocity and the deep earthquake^10^,^11^. The phase transition mechanism of materials under high pressure has shown many unique features and is still under exploration^12^,^13^,^14^.

During a pressure induced first order solid-solid transformation, usually both phases can co-exist over a certain pressure range (transition zone). The crystal structure and lattice parameters of the new phase are different from the original phase, thus, theoretically speaking internal lattice strain should be generated during the nucleation and growth of new phase to accommodate the misfit between nucleus and matrix^15^. Especially, in a displacive phase transition there would be an orientation relationship between each phase and the new phase grows epitaxially on certain lattice plane of the matrix phase, thereby the lattices of each phase on the interface may be mismatched and internal strain arises^16^. The internal strain stemmed from lattice misfit enhances the transformation potential barrier. Thereby it affects the transformation kinetics and is assumed to be responsible for many important phenomena under pressure^14^,^17^,^18^,^19^. In addition, the phase transition induced elastic strain is usually anisotropic, making the actual microscopic deviatoric stress on material higher than external applied stress and is expected to result in plastic deformation in material^19^,^20^,^21^. Namely, misfit strain could soften materials during phase transition. However, such phase transition induced elastic lattice strain has not yet been well examined experimentally under high pressure, therefore it is still an ambiguous explanation for related phenomena. Whether such internal strain exists, what are the characteristics of it, how it evolves during phase transition and how mechanical property is affected are all unresolved questions.

In the present work, using *in situ* high pressure synchrotron x-ray diffraction, we checked the compressibility of ZnO which experiences a wurtzite-rocksalt phase transition under pressure^22^,^23^,^24^, identifying the transformation induced elastic strain. Moreover, the expected strength softening during phase transition of ZnO was observed under non-hydrostatic pressure.

## Results and Discussions

**Figure 1:**
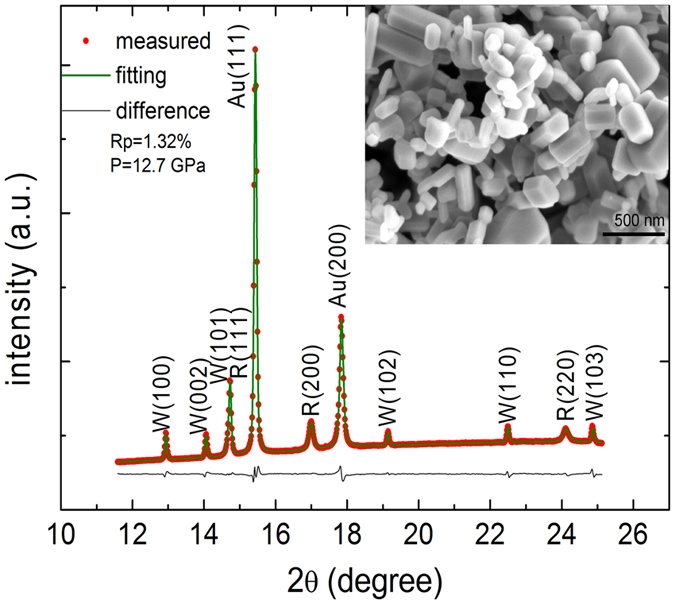
A Rietvelt refinement of the powder x-ray diffraction pattern of ZnO under 12.7 GPa. Both wurtzite and rocksalt phases are co-existed and fitted simultaneously. The inset shows the SEM image of ZnO with particle size ranges from 100 *nm* to 500 *nm*.

**Figure 2:**
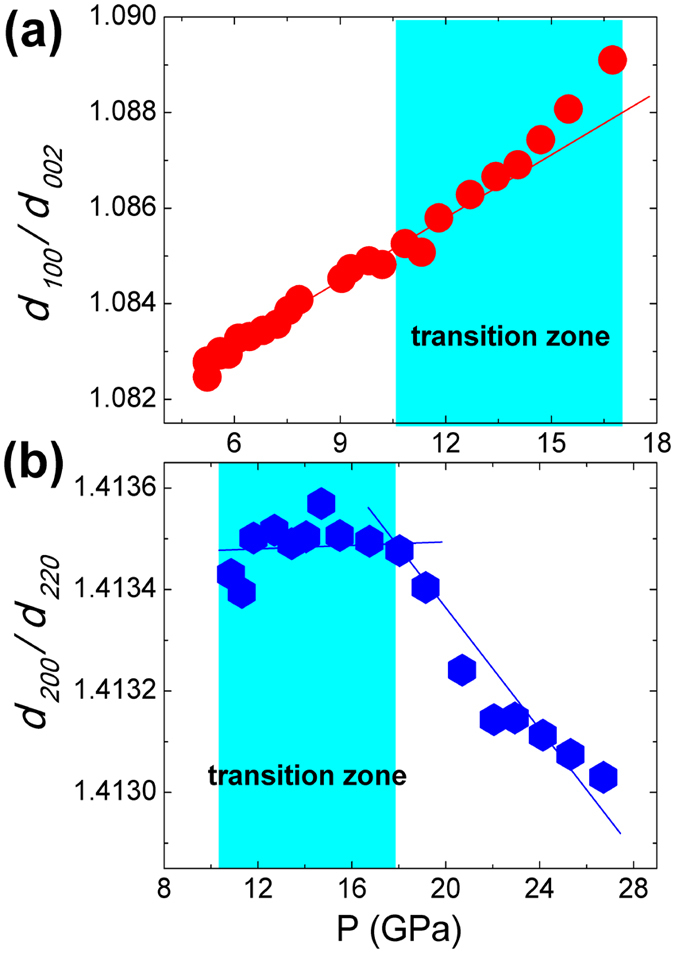
Pressure dependencies of ratio *d*_*100*_*/d*_*002*_ for wurtzite phase (**a**) and ratio *d*_*200*_*/d*_*220*_ for rocksalt phase (**b**). The straight lines guide for eyes.

In the axial x-ray diffraction (AXRD) experiments, the wurtzite-to-rocksalt transition starts from 10.1 GPa with increasing pressure. These two phases coexist over a pressure range of 10.1 GPa to 16.75 GPa, in good agreement with previous reports^23^,^24^,^25^. A typical XRD pattern and its Rietveld refinements of both wurtzite and rocksalt phases at 12.7 GPa are shown in Fig. 1. Figure 2 displays the variations of ratio *d*_*100*_*/d*_*002*_ for wurtzite phase and the ratio *d*_*200*_*/d*_*220*_ for rocksalt phase. Before phase transition the *d*_*100*_*/d*_*002*_ increases linearly with pressure, and deviates from the linear relationship in the transition zone [Fig. 2(a)], while *d*_*200*_*/d*_*220*_ decreases with pressure after the phase transition and deviates from this trend in the transition zone [Fig. 2(b)]. These observations indicate that both the wurtzite phase and rocksalt phase of ZnO are distorted in the transition zone.

**Figure 3:**
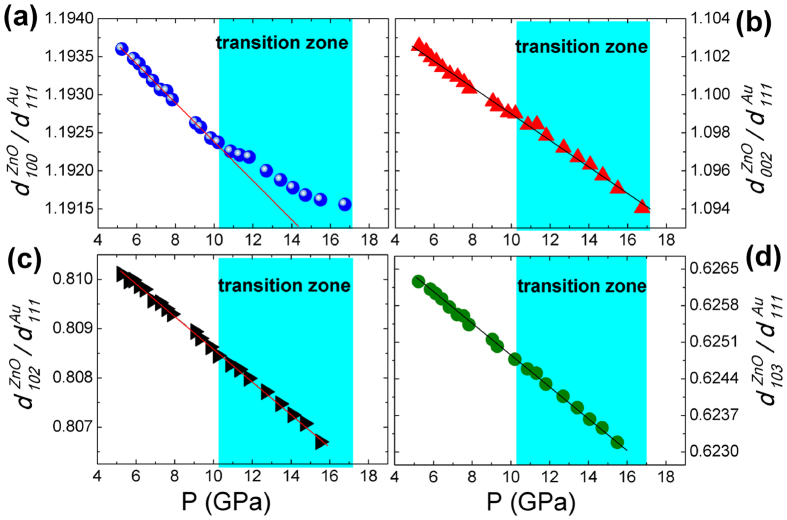
Relative value of *d*-spacings of (100) plane (**a**), (002) plane (**b**), (102) plane (**c**) and (103) plane (**d**) of wurtzite phase compared to the *d*-spacing of (111) plane of Au. The straight lines guide for eyes.

To explore the compression behavior of each lattice plane during phase transition, we take the (111) *d*-spacing of Au 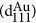 as a reference, and the ratio 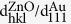
is used to represent the relative value of (h k l) *d*-spacing of ZnO compared to the (111) *d*-spacing of Au. As a common pressure standard, Au is believed to display no abnormal compression discontinuity in the pressure range in this work, so any discontinuities of the variation of 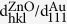 should be attributed to the unusual compression behavior of ZnO itself. Figure 3 shows the pressure evolution of ratio 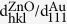 for wurtzite phase. Before the wurtzite-rocksalt phase transition,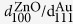, 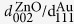, 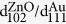 and 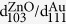 all decrease monotonically with pressure. In the transition zone, evolution of 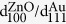 shows an abnormal change, indicating an unusual compression behavior of the *d*-spacing of (100) plane of wurtzite ZnO, while the pressure evolutions of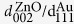, 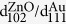 and 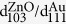 keep the linear relationships just as those before the phase transition. Furthermore, 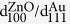
deviates to larger values from the normal linear relationship, indicating an enlarged (100) *d*-spacing of wurtzite phase, which result in that the ratio *d*_*100*_*/d*_*002*_ deviates to larger values, as seen in Fig. 2(a).

**Figure 4:**
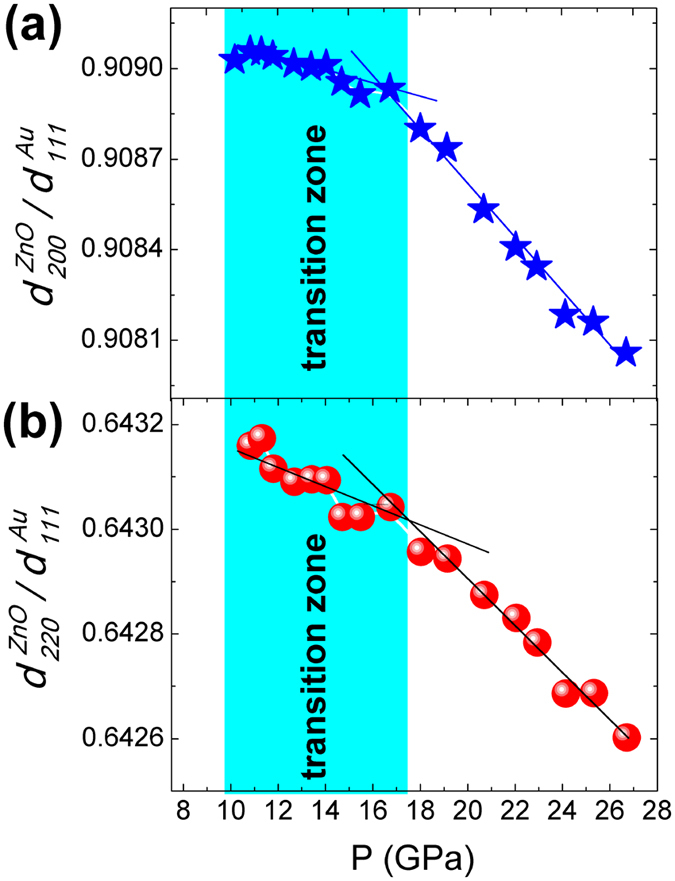
Relative value of *d*-spacings of (200) plane (**a**) and (220) plane (**b**) of rocksalt phase ZnO compared to the *d*-spacing of (111) plane of Au. The straight lines guide for eyes.

The pressure dependent 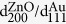 and 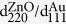 for rocksalt phase are presented in Fig. 4. After the phase transition completes, 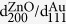 and 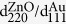 decrease approximately linearly with pressure. However, in transition zone these two ratios deviate to small values from the extrapolated line from higher pressure data, indicating an internal compressive strain on (200) plane and (220) plane of rocksalt phase. The relative deviation of 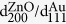 is more significant than that of 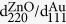, as a result the ratio *d*_*200*_*/d*_*220*_ of rocksalt phase deviates to smaller values, as seen in Fig. 2(b).

**Figure 5:**
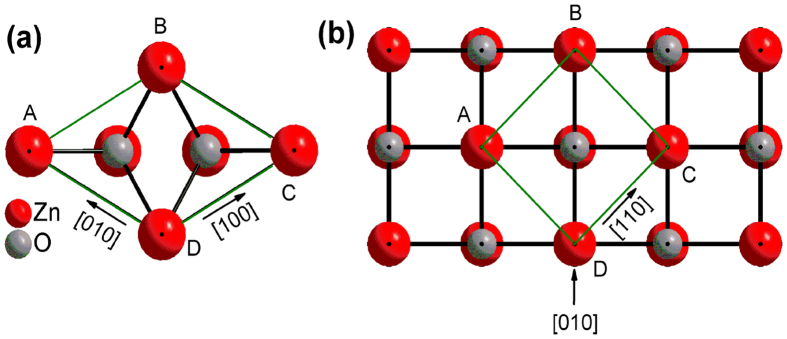
Top views of wurtzite phase (**a**) and rocksalt phase (**b**) crystal structures.

Limpijumnong *et al*.^26^ proposed that the wurtzite-rocksalt phase transition should follow the hexagonal path, in which the *c/a* ratio decreased first, and then the hexagonal angle opens up while the atoms in the center of the triangle move horizontally to the center of the square, the rocksalt phase then occurs. In this model, wurtzite phase (Fig. 5(a)) can continuously transform into rocksalt phase (Fig. 5(b)) along a simple homogeneous orthorhombic shear strain path, consequently the relative crystallographic orientation of the wurtzite and rocksalt phases with (001)_wurtzite_ || (001)_rocksalt_ is expected^26^. This phase transition model is also believed to apply to nanocrystals^27^ and has been validated by experimental observations^28^,^29^.

At the onset of the phase transition, the rocksalt phase grows epitaxially on the matrix of the wurtzite phase because of the crystallographic relationship of these two phases. When wurtzite and rocksalt phases occur as intimately mixed phases with an epitaxial relationship between them, each phase is subjected to a stress imposed by the other phase. As a result, each phase would be elastically deformed with respect to what it would be when the other phase were absent. As a result of the crystallographic relationship with (001)_wurtzite_ || (001)_rocksalt_, the (001) plane of rocksalt phase is expected to grow epitaxially on the (001) plane of wurtzite phase. In the wurtzite-rocksalt phase transition, the rhombus-shaped unit cell projection along the [001] direction in wurtzite phase transforms to a square in rocksalt phase (Fig. 5)^26^. In this process the *d*-spacing of (100) plane of wurtzite phase needs to increase to match the (110) plane of rocksalt phase, as *d*_*110*_ of rocksalt phase is larger than *d*_*100*_ of wurtzite phase. Therefore, *d*_*100*_ of wurtzite phase would be stretched by the larger lattice dimension of (110) plane of rocksalt phase, while accordingly *d*_*110*_ (or *d*_*220*_) of rocksalt phase would be compressed to match the smaller lattice dimension of (100) plane of wurtzite phase. Owing to the symmetry of rocksalt phase, *d*_*200*_ equals to 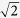
*d*_*220*_ and thus shows more obvious unusual compression behavior than *d*_*220*_ (Fig. 4), therefore the *d*_*200*_*/d*_*220*_ of rocksalt phase deviates to smaller values, as seen in Fig. 2(b).

In the initial stage of transformation, the amount of rocksalt phase is small. The lattice parameter of wurtzite phase does not deviate significantly (Fig. 3(a)) due to the relative small strained interface compared to the whole detected volume, while the deviation of lattice parameters of rocksalt phase is large (Fig. 4). For a similar reason, as the amount of the rocksalt phase is dominant near the end of transition, the deviation of lattice parameter of wurtzite phase increases and the lattice parameter deviation in rocksalt phase decreases.

**Figure 6:**
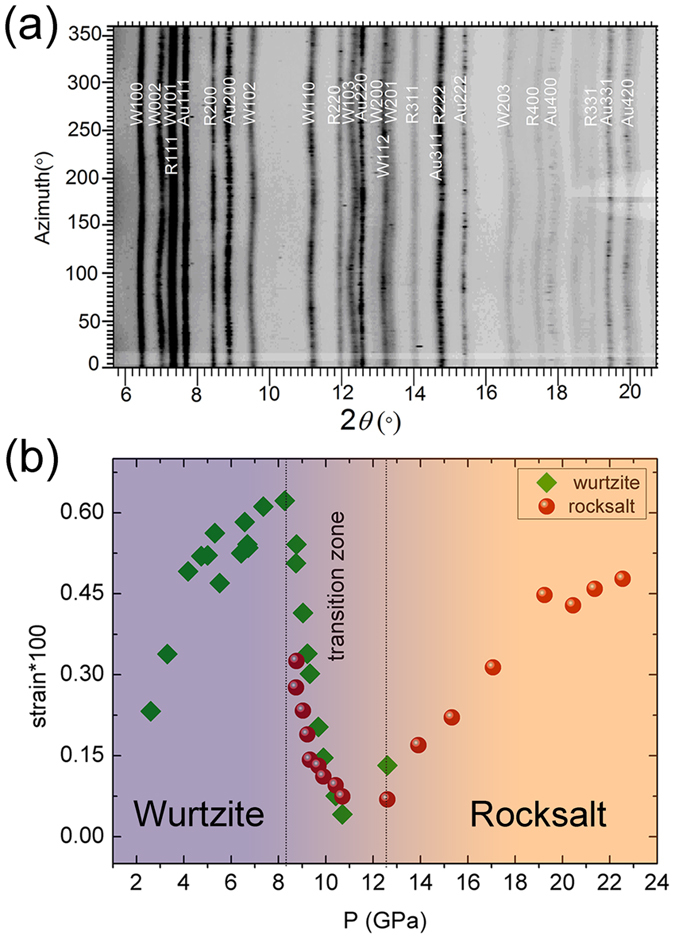
(**a**) Caked RXRD pattern of ZnO at 9.7 GPa showing detector azimuth versus 2*θ*. (**b**) Average lattice strains of wurtzite ZnO and rocksalt ZnO under pressure.

To check the influence of internal strain on the plastic property of ZnO, we derive differential elastic lattice strains for both phases of ZnO from line shifts in radial X-ray diffraction (RXRD) data collected at pressure^30^ (Fig. 6(a)). The RXRD measurements were performed under non-hydrostatic conditions. Before the wurtzite-rocksalt phase transition, the average differential strain <Q (*hkl*)>, which refer the arithmetic average of measured differential strains of all lattice planes, increases with pressure. The differential strain of rocksalt ZnO also increases with pressure when the phase transition finished. But during the phase transition, <Q (*hkl*)> of each phase ZnO decreases steeply with pressure, indicating that plastic flow is achieved^31^ (Fig. 6(b)) and the strength of each phase ZnO decreases.

As indicated in Figs 3 and 4, the internal strain on different lattice planes vary considerably, implying that the internal strain in ZnO is significantly anisotropic and leads to the development of localized shear stress. Such internal shear stress could make the actual shear stress in ZnO reach its yield strength, resulting in the deformation of ZnO. In the perspective of energy, the strain energy resulted from the lattice misfit accompanying transformation inhibits the phase transition by counteracting the chemical free energy driving force for growth. The plastic flow may relax this internal strain and enable the growth of rocksalt phase to continue^19^,^20^. Thereby, the anisotropic internal strain during phase transition could lead to the decrease of strength.

In summary, *in situ* synchrotron XRD measurements under quasi-hydrostatic conditions reveal unusual compression behaviors in ZnO during the wurtzite-rocksalt phase transition, indicating the internal elastic strain on selected lattice planes. Combined with the established phase transition model of ZnO, the observed elastic strain is believed to be caused by the lattice misfit between wurtzite phase and rocksalt phase during phase transition. Additionally, the internal elastic strain is supposed to give rise to strength softening of each phase ZnO during phase transition, which is observed in the present RXRD measurements at non-hydrostatic conditions. These results supply a strong evidence for the existence of phase transition induced elastic lattice strain under pressure and indicate that such strain could have significant affect on the physical property of material.

## Methods

*In situ* high pressure AXRD experiments were performed at room temperature, and designed to generate quasi-hydrostatic conditions by using neon as a pressure transmitting medium. Polycrystalline ZnO powder (inset in Fig. 1) with a purity of 99.99% (Aladdin Company) was pressed to a pellet with thickness about 10 *μm*, and then loaded into a 120-*μm*-diameter sample hole drilled in a rhenium gasket with 40 *μm* in thickness. Fine Au powder was mixed with the sample while a tiny ruby ball was loaded near the sample. The equation of state of Au^32^ and the ruby fluorescence shift^33^ were used for pressure calibrations. In the RXRD experiments an x-ray transparent boron-epoxy disc with 40 *μm* in thickness was used as gasket. The mixture of ZnO and gold powder was loaded into a 40-*μm*-diameter sample hole and the equation of state of Au^32^ was used for pressure calibrations. No pressure medium was used in the RXRD measurements.

The AXRD experiments were performed at the 4W2 beamline of the Beijing Synchrotron Radiation Facility (BSRF) with a wavelength of 0.6199 Å. The RXRD measurements were carried out at 16-BMD station of the High-Pressure Collaborative Access Team (HPCAT), Advanced Photon Source, Argonne National Laboratory, with a wavelength of 0.3101 Å. Diffraction patterns at various pressures were recorded with a Mar345 imaging plate detector. Collected data were analyzed for lattice parameters and differential elastic lattice strains using the programs FIT2D^34^ and MAUD^35^.

## Additional Information

**How to cite this article**: Yan, X. *et al*. Phase transition induced strain in ZnO under high pressure. *Sci. Rep.* **6**, 24958; doi: 10.1038/srep24958 (2016).
